# The relationship of foot and ankle mobility to the frontal plane projection angle in asymptomatic adults

**DOI:** 10.1186/s13047-016-0134-9

**Published:** 2016-01-25

**Authors:** Narelle Wyndow, Amy De Jong, Krystal Rial, Kylie Tucker, Natalie Collins, Bill Vicenzino, Trevor Russell, Kay Crossley

**Affiliations:** 1 Division of Physiotherapy, School of Health & Rehabilitation Sciences, The University of Queensland, St. Lucia Queensland, 4072 Australia; 2 School of Biomedical Sciences, The University of Queensland, St. Lucia Queensland, 4072 Australia; 3 Department of Mechanical Engineering, Melbourne School of Engineering, University of Melbourne, Melbourne, VIC 3010 Australia; 4 School of Allied Health, College of Science, Health and Engineering, La Trobe University, Bundoora, Vic 3086 Australia

**Keywords:** Foot, Ankle, Knee, Linear models

## Abstract

**Background:**

The frontal plane projection angle (FPPA) is frequently used as a measure of dynamic knee valgus during functional tasks, such as the single leg squat. Increased dynamic knee valgus is observed in people with knee pathologies including patellofemoral pain and anterior cruciate injury. As the foot is the primary interface with the support surface, foot and ankle mobility may affect the FPPA. This study investigated the relationship between foot and ankle mobility and the FPPA in asymptomatic adults.

**Methods:**

Thirty healthy people (aged 18–50 years) performed 5 single leg squats. Peak FPPA and FPPA excursion were determined from digital video recordings. Foot mobility was quantified as the difference in dorsal midfoot height or midfoot width, between non-weightbearing and bilateral weightbearing positions. Ankle joint dorsiflexion range was measured as the maximum distance in centimetres between the longest toe and the wall during a knee-to-wall lunge. Linear regressions with generalised estimating equations were used to examine relationships between variables.

**Results:**

Higher midfoot width mobility was associated with greater peak FPPA (*β* 0.90, *p* < 0.001, odds ratio [OR] 2.5), and FPPA excursion (*β* 0.67, *p* < 0.001, OR 1.9). Lower midfoot height mobility was associated with greater peak FPPA (*β* 0.37, *p* = 0.030, OR 1.4) and FPPA excursion (*β* 0.30, *p* = 0.020, OR 1.3). Lower ankle joint dorsiflexion was also associated with greater peak FPPA (*β* 0.61, *p* = 0.008, OR 1.8) and greater FPPA excursion (*β* 0.56, *p* < 0.001, OR 1.7).

**Conclusions:**

Foot and ankle mobility was significantly related to the FPPA during the single leg squat in healthy individuals. Specifically, higher midfoot width mobility, or lower ankle joint dorsiflexion range and midfoot height mobility, were associated with a greater FPPA. These foot mobility factors should be considered in the clinical management of knee-related disorders that are associated with a high FPPA.

## Background

Lower limb functional tests are used clinically to assess individuals at risk of lower limb pathology, and to evaluate the effect of therapeutic interventions [1]. The single leg squat is one such test that is commonly used, and can be easily implemented in the clinic. Greater dynamic knee valgus during the single leg squat, which can be measured as the frontal plane projection angle (FPPA) of the knee, consists of a combination of femoral internal rotation, hip adduction, knee flexion and knee abduction [2–4]. The FPPA measured during single leg squat is correlated with 3D kinematics of the knee during more dynamic tasks involving primarily sagittal plane motion such as running [5], thus supporting the clinical utility of this test.

During weightbearing activities, the foot interfaces with the ground [6] and so individual variations in foot and ankle mobility may affect knee motion, including the FPPA. For example, experimentally limiting the available range of ankle joint dorsiflexion during a double leg squat in asymptomatic individuals, results in an increased FPPA [7]. Structural measures of foot alignment, such as a forefoot varus deformity, have also been shown to predict a greater FPPA during a single leg jump in asymptomatic college athletes [2]. It is likely that foot and ankle mobility can influence the FPPA and hence, may be potential targets for interventions. Currently, no studies have directly investigated the relationship between ankle and foot mobility and the FPPA during a single leg squat.

A high FPPA during dynamic tasks is proposed to represent large frontal and transverse plane knee motions, which is linked to chronic aberrant knee loading. A high FPPA can discriminate those with patellofemoral pain (PFP) from those without [3]. In addition, high knee abduction moments, which are highly correlated with increased knee valgus angles [8], can predict those at risk of developing PFP and anterior cruciate ligament (ACL) injury [9, 10]. Therefore, interventions with the capacity to reduce the FPPA may be important in the management or prevention of PFP and ACL injuries.

This study aimed to determine the relationship between the FPPA during a single leg squat and mobility measures of the foot and ankle in asymptomatic individuals. We hypothesised that higher midfoot width and height mobility would be associated with a higher FPPA due to a destabilised base of support. In addition, we hypothesised that a reduced ankle joint dorsiflexion range limiting sagittal plane movement of the tibia during squatting would be associated with a higher FPPA. This study also aimed to provide baseline information against which future studies involving populations with pathology such as PFP or ACL injuries can be compared.

## Methods

### Participants

This correlational study was conducted on 30 asymptomatic participants recruited from a convenience sample of staff and students at The University of Queensland, Australia. For study inclusion, participants were required to be between 18–50 years of age, and were excluded if they had: (i) a history of foot, knee or hip surgery, (ii) current or previous pain in the knee, hip, lumbar spine or foot that had lasted longer than 3 months and/or required intervention, (iii) any neurological or systemic arthritis conditions, or (iv) an inability to understand written and spoken English. This study was approved by The University of Queensland Medical Research Ethics Committee (approval number: 2013000467), and each participant gave written informed consent prior to participation.

### Procedures

Participants’ height and weight were recorded. Participants were required to be barefoot and wear brief shorts for the assessment of lower limb function. Both legs were tested in a random order.

Coloured markers (green electrical tape approximately 15 mm wide arranged in a cross) were placed bilaterally over the anterior superior iliac spines, the midpoint of the femoral condyles, and the midpoint of the malleoli of the ankles (by examiner ADJ). Digital video recordings (Microsoft Kinect™ camera) of the frontal plane were made while participants performed the single leg squat test. The camera was placed approximately 2.0 m directly in front of the participant, at a height of approximately 0.9 m from the ground. This height aligned approximately to the level of the participants’ pelvis. Video recording were made at PAL video frame rates of approximately 25 frames per second at a resolution of 800 x 600 pixels.

Participants stood with their feet aligned in the sagittal plane, indicated by markings on the floor, and their arms folded across their chest. Participants performed five repetitions of a single leg squat on each leg. Participants were instructed to squat to a depth of 60° of knee flexion as indicated by touch of the participants buttocks to a tripod positioned behind the participant. Upon touch, participants were to immediately begin the rise to an upright single leg stance. The task was performed at a cadence of 2 s per rise and 2 s per lower as indicated by a metronome.

**Fig. 1:**
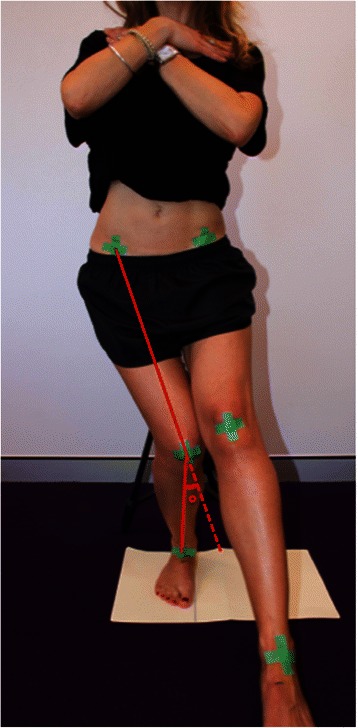
Measurement of the FPPA

The raw video footage was imported into a 2D motion analysis program (eHAB V3, NeoRehab Pty Ltd, Brisbane) for conversion to still frames and subsequent analysis. The frame at which the knee marker was at its highest point of the 2nd, 3rd and 4th squat just prior to the commencement of knee flexion was selected to indicate the top of the squat, and the frame with the lowest point of knee flexion as indicated by the knee marker, prior to the knee extending was selected as the bottom of the squat. As described by Murphy et al. [11], the eHAB software superimposes lines to connect the selected hip, knee and ankle landmarks and then calculates the FPPA (Fig. 1). Angles were measured in degrees, with a varus knee angle defined as a negative angle and a valgus knee angle defined as a positive angle [12]. The primary FPPA variables of interest were the FPPA at: (i) the lowest point of the knee marker during the squat (FPPApeak), and (ii) the difference between the FPPA when the knee markers were at their highest point just prior to knee flexion, to the maximum angle obtained at the lowest point of the squat. This measure was used to quantify the amount of knee excursion (FPPAexc). The average of three trials was obtained for analysis. The 2D FPPA measurements utilised in this study were determined to have a between-day standard error of the mean (SEM) of 0.63–0.74° for the single leg squat. Two-dimensional FPPA measures have excellent agreement with 3D motion analysis measures (ICC = 0.918) [13].

**Fig. 2:**
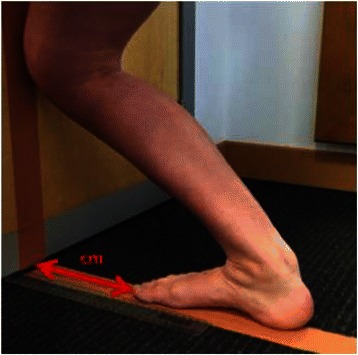
Measurement of ankle joint dorsiflexion range

To measure ankle joint dorsiflexion range, the participant aligned their 2nd toe and the bisection of their calcaneus along a line perpendicular to a wall, and the middle of their knee against the vertical projection of the perpendicular floor line (Fig. 2). They were then asked to move their heel back along this line and attempt to flex the knee forward to again touch the perpendicular wall line while keeping their foot and knee aligned in the sagittal plane. The maximum distance (in centimetres) that was achieved with the knee touching the wall and without the heel lifting was measured from the wall to their longest toe [14].

**Fig. 3:**
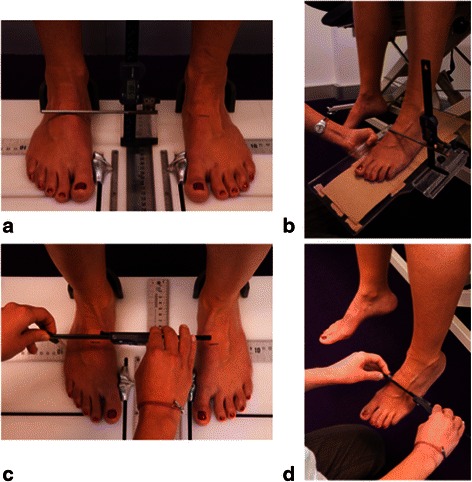
Measurement of midfoot height mobility. Midfoot height at 50 % total foot length in bilateral weightbearing (**a**). Midfoot height measurement at 50 % total foot length in non-weightbearing (**b**). Bilateral weightbearing midfoot width measurement at 50 % total foot length (**c**). Non-weightbearing midfoot width measured at 50 % total foot length (**d**)

Foot structure and mobility were measured as described by McPoil et al. [15], utilising a custom-made foot measurement platform. Initially, total foot length was obtained in bilateral lower limb weightbearing. At 50 % of the total foot length, the height of the midfoot, and midfoot width was measured (Fig. 3a and c). The participant was then assessed in a seated non-weight bearing position, with the legs hanging freely off the edge of an examination plinth, and the foot and ankle relaxed. A non-weightbearing measurement platform was positioned until it was minimally contacting the plantar surface of the foot, at which point the midfoot height measure was repeated (Fig. 3b). Midfoot width was also measured in this seated position, without the non-weightbearing platform (Fig. 3d). Foot mobility was characterised by two measures: (i) the difference in midfoot height from non-weightbearing to standing with approximately equal body weight on each foot, and (ii) the difference in midfoot width from non-weightbearing to standing with approximately equal body weight on each foot. Foot mobility measures have been shown to have high levels of intra- and inter-rater reliability [16].

### Data analysis

Linear regression models with generalised estimating equations were utilised to allow data from both limbs to be included in the same data set as potentially correlated observations between legs from the same person are accounted for. Two separate models were used to examine the association between the input variables of ankle joint dorsiflexion range, midfoot width mobility, midfoot height mobility, height and weight, and each of the FPPA indices (FPPApeak, FPPAexc). Sex was entered as a factor into each model. Betas (*β*), *p* values and odds ratios (OR) with 95 % confidence intervals (CI) are reported for each model. In order to more easily interpret the factors associated with a higher FPPA (more valgus), the signs for the input variables were converted if required. For ankle and midfoot height mobility, a positive OR indicates lower mobility and is associated with a greater FPPA. For midfoot mobility, a positive OR indicates higher mobility and is associated with a greater FPPA. Significance was set at *p* < 0.050. All analyses were performed with IBM SPSS version 22 (Chicago Illinois, USA).

## Results

Of the 32 individuals who participated in this study, 2 were excluded due to incomplete data sets, leaving 30 individuals (60 limbs) in the final analysis (19 (67 %) women; mean (SD) age 22 (3) years; mean (SD) BMI 21.7 (2.9) kg/m^2^).

Means and standard deviations for each measure are presented in Table 1. Based on Pearson’s product moments, there were no significant correlations between the midfoot and ankle variables and thus, all measures were included in the same model.

**Table 1:** Means and standard deviations of each of the measures

*Measure*	*N limbs*	*Mean (SD)*
Midfoot width mobility (mm)	60	10 (3)
Midfoot height mobility (mm)	60	15 (3)
Ankle joint dorsiflexion (cm)	60	10.7 (3.4)
FPPApeak (°)	58	4 (7)
FPPAexc (°)	60	1 (5)

*N limbs* number of limbs; *FPPASq* the FPPA during a single leg squat; *FPPAexc* single leg squat excursion; *SD* standard deviation

During the single leg squat, higher midfoot width mobility accounted for the largest contribution to a greater (more valgus) FPPA, while lower ankle joint dorsiflexion range and lower midfoot height mobility were also associated with a greater (more valgus) FPPA (midfoot width mobility: *β* 0.90, *p* < 0.001, OR 2.5, 95 % CI 1.6 to 3.7; ankle joint dorsiflexion: *β* 0.61, *p* = 0.008 OR 1.8, 95 % CI 1.2 to 2.9; midfoot height mobility: *β* 0.44, *p* = 0.030, OR 1.5, 95 % CI 1.1 to 2.3). Similar trends for each foot mobility variable on FPPA excursion during the single leg squat were observed (Table 2).

**Table 2:** Odds ratios, 95 % confidence intervals and *p* values for foot and ankle factors associated with the FPPA

	FPPApeak		FPPAexc	
	*OR(95 % CI)*	*P-value*	*OR(95 % CI)*	*P-value*
Midfoot width mobility	2.4(1.63–3.61)	<.001	1.9(1.51–2.51)	<0.001
Ankle joint dorsiflexion	1.8(1.18–2.90)	0.008	1.7(1.32–2.31)	<0.001
Midfoot height mobility	1.5(1.04–2.29)	0.030	1.4(1.04–1.76)	0.020

For midfoot width mobility, a greater OR indicates that higher midfoot mobility contributes to a greater FPPA. A greater OR for ankle joint dorsiflexion and midfoot height mobility indicates that a lower range of ankle or midfoot height mobility contributes to a greater FPPA

*FPPApeak* the peak FPPA during a single leg squat; *FPPAexc* single leg squat excursion; *OR* odds ratio; *CI* confidence interval

## Discussion

Higher frontal plane foot mobility and lower sagittal plane mobility, specifically higher midfoot width mobility and lower midfoot height mobility, were significantly associated with greater FPPA (or greater valgus) during the single leg squat. Lower ankle joint dorsiflexion range was also significantly associated with greater FPPA during the squat. As a greater FPPA is associated with, and is likely to be a risk factor for, the development of PFP and anterior cruciate injury [9, 17, 18], the results of this study highlight the possibility of addressing these foot and ankle mobility factors for the prevention and management of these conditions.

Individuals with higher midfoot mobility were 2.4 times more likely to have a greater peak FPPA and 1.9 times more likely to have greater FPPA excursion during single leg squat. The association between higher frontal plane mobility at the midfoot and hip (peak FPPA) during single leg squat might provide a basis for exploring mechanisms through which physical treatments aimed at the foot improve knee or hip pain. For example, limiting motion of the midfoot with foot orthoses [19] might explain why those with PFP and higher midfoot mobility are more likely to experience positive outcomes with foot orthoses [20, 21], conceivably through a reduction in the FPPA [22].

Lower sagittal plane motions at the ankle joint and, to a lesser extent, at the midfoot (midfoot height mobility), were also significantly associated with greater FPPA during single leg squat. This finding with regards to ankle joint dorsiflexion range is consistent with previous findings in healthy controls [7]. Our results confirm that lower ankle joint dorsiflexion range is related to more dynamic knee valgus during single leg squat in asymptomatic adults. As structural restriction of ankle joint dorsiflexion range limits the anterior translation of the tibia over the fixed foot during dynamic tasks, it is possible that this is compensated for by foot pronation in order to utilise the dorsiflexion component of subtalar and midtarsal joint motion [23, 24]. This compensatory strategy will inherently involve motion in other planes due to the triplanar axis of the subtalar and midtarsal joints [25], and may contribute to transverse and frontal plane tibial motions, potentially increasing the FPPA. Alternatively, individuals with lower ankle dorsiflexion may utilise different hip strategies, such as increased hip adduction and internal rotation, to achieve a greater depth of squat. One study has demonstrated that foot orthoses can reduce hip adduction during a step-up task in those with lower ankle joint dorsiflexion range [22]. It is possible that the foot orthoses employed in this previous study may have elevated the heel sufficiently to improve the available ankle joint sagittal plane range of motion.

Lower midfoot height mobility was significantly associated with greater FPPA. This finding was surprising, as higher midfoot mobility would be expected to present as both greater midfoot width and midfoot lowering with weightbearing. However, as highlighted by Nester et al. [25], the joints of the foot show incomplete evidence of coupling, and there is high person to person variability in joint coupling. It is plausible that in the current sample, lower midfoot height mobility restricted sagittal plane compensatory motion at the midtarsal joint [24], and induced similar compensations as reduced ankle joint dorsiflexion. This proposition requires further investigation.

A greater FPPA during the single leg squat is a feature of people with PFP [3]. High knee valgus moments during a drop vertical jump task, which is a similar measure to the FPPA, can predict those at higher risk of ACL injury and patellofemoral pain [9, 10]. We observed that foot mobility measures are associated with increased FPPA during a single leg functional task. Clinical interventions directed towards ameliorating the negative effect of these foot and ankle factors on the FPPA may be important in injury management or prevention of these conditions. For example, foot orthoses can immediately reduce hip adduction and knee internal rotation (both components of the FPPA) during a step-up task in healthy individuals [22], while a full length 5° medial foot wedge reduces hip adduction, hip internal rotation and knee valgus during a single leg squat [26]. Strengthening exercises of the foot intrinsic muscles in those with high midfoot mobility may also provide functional performance benefits [27, 28]. In those with reduced ankle joint dorsiflexion range, common clinical interventions for this presentation, such as calf stretching and/or the use of heel lifts, may minimise the negative impact of this feature on lower limb function.

This study focused on foot mobility measures, therefore specific forefoot alignment measures, such as the forefoot varus angle, were not included in this study. However, midfoot mobility measures are composite measures of motion in all foot regions [29], and will partially account for major structural deviations at the forefoot. As static forefoot alignment can predict greater FPPA during double limb squats and jumps [2], and greater hip internal rotation during a single leg squat [30], future studies may benefit from the inclusion of forefoot alignment. In addition, this study only focused on distal factors, so proximal factors, such as range of motion and strength of the hip joint [1, 31], should be included in a more comprehensive perspective of proximal and distal contributors to the FPPA. Finally, the results cannot be generalised to clinical populations. The presence of pain may alter the compensatory strategies employed [32] in response to changes in foot and ankle mobility. For example, those with chronic PFP may avoid deep knee flexion and adduction by employing more hip flexion, as has been shown during stair ascent [33]. Further studies are warranted to determine the relationship between foot and ankle mobility and specific lower limb pathologies.

## Conclusion

Higher midfoot mobility was significantly associated with greater FPPA during single leg squat, as was lower ankle joint dorsiflexion and sagittal plane midfoot mobility. Addressing these variables may result in a more optimal FPPA and potentially be important in the prevention or management of lower limb injury.

## Abbreviations

FPPA: frontal plane projection angle
cm: centimetres
OR: odds ratio
PFP: patellofemoral pain
ACL: anterior cruciate ligament
FPPApeak: peak frontal plane projection angle
FPPAexc: excursion of the frontal plane projection angle
*β*: beta
CI: confidence interval
SD: standard deviation
BMI: body mass index
Kg/m^2^: kilograms per square metre

## References

[1] Crossley KM, Zhang WJ, Schache AG, Bryant A, Cowan SM (2011). Performance on the single-leg squat task indicates hip abductor muscle function. Am J Sports Med.

[2] Bittencourt NF, Ocarino JM, Mendonca LD, Hewett TE, Fonseca ST (2012). Foot and hip contributions to high frontal plane knee projection angle in athletes: a classification and regression tree approach. J Orthop Sports Phys Ther.

[3] Willson JD, Davis IS (2008). Utility of the frontal plane projection angle in females with patellofemoral pain. J Orthop Sports Phys Ther.

[4] Powers CM (2010). The influence of abnormal hip mechanics on knee injury: a biomechanical perspective. J Orthop Sports Phys Ther.

[5] Whatman C, Hing W, Hume P (2011). Kinematics during lower extremity functional screening tests-are they reliable and related to jogging?. Phys Ther Sport.

[6] Dugan SA, Bhat KP (2005). Biomechanics and analysis of running gait. Phys Med Rehabil Clin N Am.

[7] Macrum E, Bell DR, Boling M, Lewek M, Padua D (2012). Effect of limiting ankle-dorsiflexion range of motion on lower extremity kinematics and muscle-activation patterns during a squat. J Sport Rehabil.

[8] Myer GD, Ford KR, Khoury J, Succop P, Hewett TE (2010). Clinical correlates to laboratory measures for use in non-contact anterior cruciate ligament injury risk prediction algorithm. Clin Biomech (Bristol, Avon).

[9] Myer GD, Ford KR, Di Stasi SL, Barber Foss KD, Micheli LJ, Hewett TE (2015). High knee abduction moments are common risk factors for patellofemoral pain (PFP) and anterior cruciate ligament (ACL) injury in girls: Is PFP itself a predictor for subsequent ACL injury?. Br J Sports Med.

[10] Myer GD, Ford KR, Khoury J, Succop P, Hewett TE (2010). Development and validation of a clinic-based prediction tool to identify female athletes at high risk for anterior cruciate ligament injury. Am J Sports Med.

[11] Murphy M, Hides J, Russell T (2013). A Digital Photographic Technique for Knee Range of Motion Measurement: Performance in a Total Knee Arthroplasty Clinical Population. Open J Orthop..

[12] Munro A, Herrington L, Carolan M (2012). Reliability of 2-dimensional video assessment of frontal-plane dynamic knee valgus during common athletic screening tasks. J Sport Rehabil.

[13] Mizner RL, Chmielewski TL, Toepke JJ, Tofte KB (2012). Comparison of 2-dimensional measurement techniques for predicting knee angle and moment during a drop vertical jump. Clin J Sport Med.

[14] Bennell KL, Talbot RC, Wajswelner H, Techovanich W, Kelly DH, Hall AJ (1998). Intra-rater and inter-rater reliability of a weight-bearing lunge measure of ankle dorsiflexion. Aust J Physiother.

[15] McPoil TG, Cornwall MW, Medoff L, Vicenzino B, Fosberg KK, Hilz D (2009). Arch height change during sit-to-stand: an alternative for the navicular drop test. J Foot Ankle Res..

[16] McPoil TG, Vicenzino B, Cornwall MW, Collins N, Warren M (2009). Reliability and normative values for the foot mobility magnitude: a composite measure of vertical and medial-lateral mobility of the midfoot. J Foot Ankle Res..

[17] Hewett TE, Myer GD, Ford KR, Heidt RS, Colosimo AJ, McLean SG (2005). Biomechanical measures of neuromuscular control and valgus loading of the knee predict anterior cruciate ligament injury risk in female athletes: a prospective study. Am J Sports Med.

[18] Myer GD, Ford KR, Barber Foss KD, Goodman A, Ceasar A, Rauh MJ (2010). The incidence and potential pathomechanics of patellofemoral pain in female athletes. Clin Biomech (Bristol, Avon).

[19] Sinclair J, Isherwood J, Taylor PJ (2015). The effects of orthotic intervention on multisegment foot kinematics and plantar fascia strain in recreational runners. J Appl Biomech.

[20] Mills K, Blanch P, Dev P, Martin M, Vicenzino B (2012). A randomised control trial of short term efficacy of in-shoe foot orthoses compared with a wait and see policy for anterior knee pain and the role of foot mobility. Br J Sports Med.

[21] Vicenzino B, Collins N, Cleland J, McPoil T (2010). A clinical prediction rule for identifying patients with patellofemoral pain who are likely to benefit from foot orthoses: a preliminary determination. Br J Sports Med.

[22] Lack S, Barton C, Malliaras P, Twycross-Lewis R, Woledge R, Morrissey D (2014). The effect of anti-pronation foot orthoses on hip and knee kinematics and muscle activity during a functional step-up task in healthy individuals: A laboratory study. Clin Biomech (Bristol, Avon)..

[23] Jung DY, Koh EK, Kwon OY, Yi CH, Oh JS, Weon JH (2009). Effect of medial arch support on displacement of the myotendinous junction of the gastrocnemius during standing wall stretching. J Orthop Sports Phys Ther.

[24] Chizewski MG, Chiu LZ (2012). Contribution of calcaneal and leg segment rotations to ankle joint dorsiflexion in a weight-bearing task. Gait Posture.

[25] Nester CJ, Jarvis HL, Jones RK, Bowden PD, Liu A (2014). Movement of the human foot in 100 pain free individuals aged 18–45: implications for understanding normal foot function. J Foot Ankle Res.

[26] Joseph MF, Holsing KL, Tiberio D (2014). Lower extremity kinematics of a single-leg squat with an orthotic in male and female collegiate athletes. J Appl Biomech.

[27] Mulligan EP, Cook PG (2013). Effect of plantar intrinsic muscle training on medial longitudinal arch morphology and dynamic function. Man Ther.

[28] Moon DC, Kim K, Lee SK (2014). Immediate Effect of Short-foot Exercise on Dynamic Balance of Subjects with Excessively Pronated Feet. J Phys Ther Sci.

[29] Nester CJ, Hutchins S, Bowker P (2001). Effect of foot orthoses on rearfoot complex kinematics during walking gait. Foot Ankle Int.

[30] Scattone Silva R, Maciel CD, Serrao FV (2015). The effects of forefoot varus on hip and knee kinematics during single-leg squat. Man Ther.

[31] Cashman GE (2012). The effect of weak hip abductors or external rotators on knee valgus kinematics in healthy subjects: a systematic review. J Sport Rehabil.

[32] Hodges PW, Tucker K (2011). Moving differently in pain: a new theory to explain the adaptation to pain. Pain..

[33] Fok LA, Schache AG, Crossley KM, Lin YC, Pandy MG (2013). Patellofemoral joint loading during stair ambulation in people with patellofemoral osteoarthritis. Arthritis Rheum.

